# Pain and suicidality in children and adolescents: a longitudinal population-based study

**DOI:** 10.1007/s00787-022-01963-2

**Published:** 2022-03-02

**Authors:** Verena Hinze, Anke Karl, Tamsin Ford, Bergljot Gjelsvik

**Affiliations:** 1 Department of Psychiatry, University of Oxford, Warneford Lane, Oxford, OX3 7JX UK; 2 Department of Psychology, University of Exeter, Exeter, UK; 3 Department of Psychiatry, University of Cambridge, Cambridge, UK; 4 Department of Psychology, University of Oslo, Oslo, Norway

**Keywords:** Adolescents, Children, Pain, Suicidality, Longitudinal

## Abstract

Suicidality is a common public health concern in young people. Previous research has highlighted pain as a key correlate of suicidality in young people. However, the long-term experience of pain may vary between individuals, and the relationship between distinct pain trajectories and suicidality is poorly understood. This study aims to describe the number and nature of distinct pain trajectories, their demographic and clinical correlates, including baseline suicidality, and whether identified pain trajectories may predict future suicidality. Secondary data analyses were performed, using longitudinal data from the British Child and Adolescent Mental Health Survey (*N* = 7977), collected at five timepoints between 2004 and 2007 on a population-based sample of UK youth (5–16 years). Data were collected from up to three respondents (parents, teachers, and 11 + year-olds). Latent Class Growth Analysis was used to identify distinct pain trajectories, explore predictors of these trajectories, and establish whether trajectories predicted future suicidality. We identified the following four pain trajectories: increasing (33.6%), decreasing (4.5%), persistent/recurrent probability of pain (15.7%), and no pain (46.2%). Pain trajectories were associated with unique demographic and clinical correlates. Only the persistent/recurrent (vs. no-pain) trajectory was predicted by baseline suicidality (aOR = 2.24; 95% bootstrap-CI = 1.59–3.26). Furthermore, the persistent/recurrent trajectory predicted future suicidality (aOR = 1.03, 95% bootstrap-CI = 1.01–1.06), after controlling for baseline suicidality, psychiatric disorder, age, and gender. Findings provide a better understanding of correlates associated with distinct pain trajectories and long-term risk of suicidality in young people, suggesting a bidirectional pain–suicidality association and emphasising the need of targeted support for young people with persistent/recurrent pain.

**Supplementary Information:**

The online version contains supplementary material available at 10.1007/s00787-022-01963-2.

## Introduction

Death by suicide was the leading cause of death in 5- to 19-year-olds in England and Wales in 2020 [1]. For each suicide, far more young people think about suicide and self-harm (29.9%) or engage in self-harm (9.7%), together referred to as ‘suicidality’ [2, 3]. Self-harm is defined as intentional self-injury or self-poisoning, regardless of suicidal intent [4]. This definition is consistent with the UK national guidelines on self-harm [5] and the view that suicidal intent is a dimensional phenomenon [3, 6]. Indeed, self-harm often re-occurs in young people and is associated with substantial suffering [7, 8] and risk of future death by suicide [9]. Hence, the identification of youth at-risk of suicidality is vital.

One factor that increases suicidal vulnerability in adults is physical pain (‘pain’), regardless of its type [10]. Pain is a sensory *and* emotional experience [11], which may explain the frequent comorbidity with psychiatric disorders [12] and elevated risk of future psychopathology [13]. Likewise, psychopathology increases the risk of persistent/recurrent paediatric pain [14], suggesting a bidirectional relationship between pain and psychopathology. Whilst emerging research suggests that pain may double the risk of suicidality in young people, and possibly predict these outcomes longitudinally, such research is sparse and inclusive [15].

Pain is highly prevalent in young people (11–38%), especially headaches (8–83%) and abdominal pain (4–53% [16]). Whilst most young people may experience short-term pains, for others pain may reoccur and persist into adulthood with a significant life impact [14, 17]. As temporal manifestations of pain may vary between individuals, it is crucial to learn which pain trajectories may increase risk of future suicidality to aid targeted support.

Three developmental trajectories for common pain problems in 12- to 17-year-olds have previously been identified as follows: none-to-minimal, sporadic, and frequent pain [18]. Yet, it remains unknown which pain trajectories may drive the pain-suicidality association in young people, and whether shared correlates of pain and suicidality may predict pain trajectories.

Previously child-specific (e.g., inhibitory control deficits), parental (e.g., parental distress) and contextual factors (particularly adverse childhood experiences) were proposed to interact to influence the progression of pain and depression in young people [19]. Similar factors have been identified as correlates of adolescent self-harm and suicide [3] and will, therefore, be considered here as shared ‘clinical’ correlates of pain and suicidality in young people. Important shared ‘demographic’ correlates include adolescent age [20, 21] (adolescence = 10–19 years [22]) and female gender [8, 16]. Whether these shared demographic and clinical correlates may be associated with distinct pain trajectories, and which pain trajectories may be associated with subsequent suicidality in young people remains unclear. Such knowledge is crucial to develop targeted interventions.

We describe the number and nature of distinct temporal pain trajectories in UK youth. We explored predictors of pain trajectories, including baseline suicidality, and how these trajectories relate to future suicidality, aiming to explore a bidirectional relationship between pain and suicidality. We investigated three hypotheses:

First, individual variability in pain can be captured with up to the following five distinct pain trajectories: low, persistent/recurrent, increasing, decreasing, and changes in the probability of pain across time.

Second, pain trajectories can be predicted by shared demographic (age & gender) and clinical correlates (suicidality, psychiatric disorder, childhood trauma, parental distress, inhibitory control deficits, and peer problems) in 2004.

Third, pain trajectories are associated with suicidality in 2007, even after adjustment for baseline suicidality, psychiatric disorder, age, and gender.

## Methods

### Participants

**Fig. 1:**
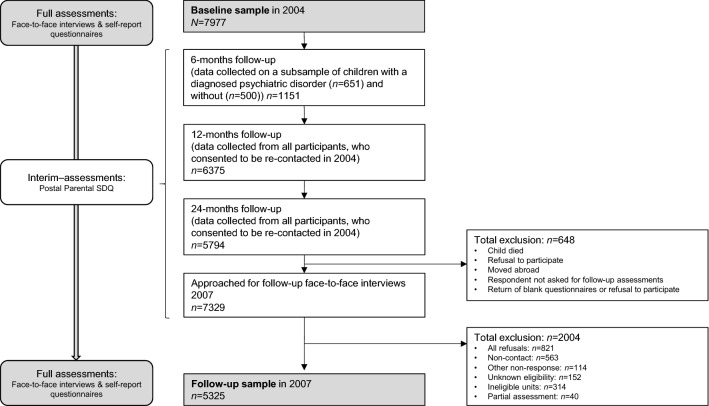
Participant flow-chart for the British Child and Adolescent Mental Health Survey (2004–2007)

Secondary data analyses were performed using longitudinal data of the British Child and Adolescent Mental Health Survey [BCAMHS] between 2004 [23] and 2007 (see Fig. 1 & Supplement 1 [24, 25]). This survey was initiated by the Department of Health and the Scottish Government and currently provides the best available, longitudinal data on the mental health of young people in Great Britain. BCAMHS was granted ethical approvals by the Medical Research Ethics Committee; given the analysis of data available via the UK Data Archive, no further ethical permission was required. Informed consent was obtained from all respondents prior to participation in this study.

### Measures

#### Demographics

We used baseline information on the child’s age (grouped as follows: 5–7, 8–10, 11–13, and 14–16 years), gender (boys vs. girls) and ethnicity (categorised as follows: Black, Indian, Pakistani/Bangladeshi, White, and Other) to describe the study sample.

#### Suicidality

In 2004 and 2007, suicidality was assessed as part of two distinct modules via parental report and self-report (11 + year-olds [23, 25]). For young people with depressive symptoms, four questions were used to assess thoughts about suicide and self-harm and self-harm behaviours in the past 4 weeks when they felt depressed and lifetime self-harm: “During the period when NAME CHILD was sad, irritable or lacking in interest… a) did s/he think about death a lot?, b) did s/he ever talk about harming himself/herself or killing himself/herself?, c) did s/he ever try to harm himself/herself or kill himself/ herself, and if not d) Over the whole of his/her lifetime has s/he ever tried to harm himself/herself or kill himself/herself?”. In the absence of depressive symptoms, a separate set of items was provided, enquiring about self-harm thoughts and behaviours in the past 4 weeks and lifetime self-harm: a) “Over the past 4 weeks, has s/he talked about deliberately harming or hurting himself/herself?”, b) Over the past 4 weeks, has s/he ever tried to harm or hurt himself/herself?”, and if not c) “Over the whole of his/her lifetime, has s/he ever tried to harm or hurt himself/herself?”. All items were scored with ‘Yes’ or ‘No’ [23, 25].

To create a single ‘Suicidality’ variable, we combined all available data as follows: coded as ‘present’, if at least one item across both modules and informants was answered with ‘Yes’ and otherwise coded as ‘absent’. Hence, ‘Suicidality’ reflects different manifestations of suicidal distress irrespective of suicidal intent and is consistent with the view that suicidal intent is a dimensional phenomenon [6]. We found a ‘fair’ inter-rater agreement between parental and young person’s reports of suicidality in 2004 (Cohen’s kappa = 0.33, 95%CI = 0.27–0.38) and 2007 (Cohen’s kappa = 0.42, 95%CI = 0.37–0.48), which aligns with previous research [26].

#### Pain

Pain in the past 6 months was measured via parental report, using an item on the Strengths and Difficulties Questionnaire [SDQ] [27]: “[The child] *Often complaints of headaches, stomach aches or sickness*” with the response options ‘Not true’ (pain coded ‘absent’), ‘Somewhat true’ and ‘Certainly true’ (for both responses: pain coded ‘present’). This binary ‘Pain’ variable correlated well with parental reports of the child’s related health conditions, including stomach/abdominal pains and migraines/severe headaches, which were assessed at baseline, by giving parents a list of common health problems or conditions and asking them to select any problem or condition that their child has (n_(with a pain problem or condition)_ = 702, 8.8%; Spearman’s rho = 0.35, p < 0.001), with considerable agreement between measurement tools (percentage agreement: 78%, 95%CI = [0.77;0.78]; Cohen’s kappa = 0.28, 95%CI = [0.26;0.30]; specificity = 0.78, 95%CI = [0.77;0.79]; sensitivity = 0.76, 95%CI = [0.73;0.79]). Consistent with previous research [28], this finding suggests convergent validity. Parental reports of their child’s pain in the past six months were collected in 2004, and at 6-, 12-, 24-months follow-up and in 2007 [23–25].

#### Clinical correlates

Clinical correlates measured in 2004 include the following: psychiatric disorder (Development and Well-Being Assessment [DAWBA] [29], multi-informant (parents, teachers, and 11-plus)), exposure to childhood trauma (DAWBA ‘Post Traumatic Stress Disorder’ module, parental report), parental distress (‘General Health Questionnaire’ [30], parental report), inhibitory control deficits and peer problems (SDQ subscales [27], parental report). All correlates were dichotomised using common cut-off thresholds. Supplement 1 provides detailed information on the measures, psychometrics, and cut-off scores.

### Statistical analyses

**Fig. 2:**
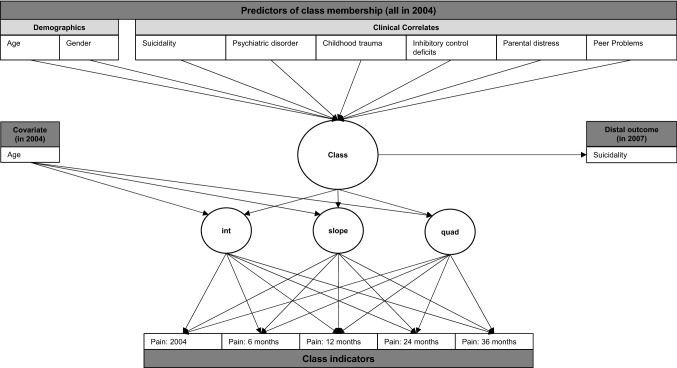
Conceptual Model. Note. The growth estimates (intercept, linear and quadratic slope) are controlled for the effects of baseline age. The association between pain class membership and suicidality is hypothesised to remain significant, after controlling for baseline suicidality, psychiatric disorder, age, and gender. The classes refer to an increasing, decreasing, persistent/recurrent probability of pain and no pain. *Int* intercept, *slope* linear slope, *quad* quadratic slope

Statistical analyses were performed in R, version 3.6.2 [31] and Mplus, version 8.5 [32]. We used two-sided contrasts with *p* < 0.05 and 95% confidence intervals to indicate statistical significance. Using Fisher’s test, we explored cross-sectional and longitudinal associations between pain and suicidality. Latent Class Growth Analysis was used to explore the number and nature of distinct temporal pain trajectories (i.e., classes) [33], using the manual Vermunt three-step approach [34]. Models were estimated, using robust, full information maximum likelihood estimation to address missingness and account for non-normality [32, 35]. Model identification was based on recommended goodness-of-fit criteria [36], successful convergence, class interpretability and class size [33], which is consistent with the ‘Guidelines for Reporting on Latent Trajectory Studies’ [GRoLTS checklist] [37]. Multinominal, multivariable regression analyses were used to a) explore whether shared correlates in 2004 predicted class membership, and b) whether class membership predicted suicidality in 2007, after adjustment for baseline suicidality, psychiatric disorder, age, and gender. The Wald-test of parameter constraints was used to reveal significant threshold differences between classes, followed-up with pairwise comparisons (reference: ‘no pain’). P-values were adjusted using Bonferroni correction, and 95% bias-corrected bootstrap confidence intervals [95%BCI] were computed to reveal the robustness of the results across 1000 bootstrap samples. Figure 2 shows the conceptual model and Supplement 2 provides information on the statistical analyses.

## Results

### Participant characteristics

In 2004, 7977 families provided data on young people aged 5 to 16 years (*M* = 10.5 years, *SD* = 3.4), with an equal gender distribution (girls: *n* = 3866; 48.5%; Table 1). Most young people identified as ‘White’ (*n* = 6920, 86.8%), and 582 (7.3%) young people reported suicidality. Pain in the past six months was reported for 2169 (27.2%) young people. Subsequent pain data were collected at 6-months (*n* = 1151; 33.7% reported pain), 12-months (*n* = 6375; 27.3% reported pain), and 24-months (*n* = 5794; 27.2% reported pain) follow-up, and in 2007 (*n* = 5325; 31.5% reported pain; Fig. S1). Overall, 7935 (99.5%) families provided data on pain at least once. Of those, 967 (12.2%) families provided data on pain once, 795 (10%) twice, 1511 (19%) three times, 3930 (49.5%) four times and 732 (9.2%) five times.

**Table 1:** Participant characteristics

		Interim assessments			
	Baseline (2004)	6-months	12-months	24-months	Follow-up (2007)
Variable	(*N* = 7977)	(*n* = 1151)^a^	(*n* = 6375)^b^	(*n* = 5794)^c^	(*n* = 5325)^d^
Demographics					
Age, M (SD)*	10.54 (3.40)				13.44 (3.32)
Group 1, n (%)	5–7 years: 1920 (24.1)				< 8–10 years: 1297 (24.4)
Group 2, n (%)	8–10 years: 2005 (25.1)				11–13 years: 1390 (26.1)
Group 3, n (%)	11–13 years: 2130 (26.7)				14–16 years: 1460 (27.4)
Group 4, n (%)	14–16 years: 1922 (24.1)				17–19 years: 1178 (22.1)
Girls, n (%)	3866 (48.5)				2580 (48.5)
Ethnicity*					
Black, n (%)	198 (2.5)^e^				97 (1.8)^e^
Indian, n (%)	199 (2.5)^e^				103 (1.9)^e^
Pakistani/ Bangladeshi, n (%)	307 (3.9)^e^				136 (2.6)^e^
White, n (%)	6920 (86.8)^e^				4785 (89.9)^e^
Other, n (%)	349 (4.4)^e^				200 (3.8)^e^
Suicidality					
Suicidality, n (%)*	582 (7.3)^f^				539 (10.1)^h^
Only thoughts, n (%)*	178 (2.2)^g^				111 (2.1)^h^
Only behaviours, n (%)	277 (3.5)^f^				324 (6.1)^h^
Self-harm thoughts and behaviours, n (%)	127 (1.6)^f^				104 (2.0)^h^
Pain					
Pain in the past six months, n (%)*	2169 (27.2)^i^	388 (33.7)^j^	1743 (27.3)^k^	1578 (27.2)^l^	1679 (31.5)^m^
Predictors of class membership					
Psychiatric disorder, n (%)*	764 (9.6)				
Childhood trauma, n (%)*	1701 (21.3)^n^				
Inhibitory control deficits, n (%)*	1518 (19.0)^o^				
Parental distress, n (%)*	1747 (21.9)^p^				
Peer problems, n (%)*	863 (10.8)^q^				

Suicidality = Computed across both modules and informants. Missing data for: a = 6826, b = 1602, c = 2183, d = 2652, e = 4, f = 98, g = 100, h = 22, i = 46, j = 3, k = 35, l = 23, m = 45, n = 187, o = 52, p = 241, q = 42. ‘*’ = Significant baseline differences between participants with and without follow-up data. Empty cells = Variables were not collected and/or used for the present analyses

In 2007, the sample consisted of 5325 families of young people aged 7 to 19 years, suggesting a drop out of 33 per cent (*n* = 2652) between 2004 and 2007. The proportion of missing data per assessment wave was low (2004: < 3%; interim assessments: < 1%; 2007: < 1%). Participants without three-year follow-up data were older, more often identified as non-White, and reported higher levels of baseline pain, suicidality and all other correlates (Table 1).

### Bivariate associations

At baseline, pain was positively associated with age (rho = 0.09, p < 0.01) and gender (OR = 1.48, 95%CI = 1.34–1.64), showing a higher likelihood of pain in girls than in boys. Furthermore, baseline pain was associated with an increased likelihood of baseline suicidality (OR = 2.75, 95%CI = 2.31–3.28), psychiatric disorder (OR = 2.73, 95%CI = 2.34–3.19), childhood trauma (OR = 1.55, 95%CI = 1.37–1.74), inhibitory control deficits (OR = 1.80, 95%CI = 1.60–2.04), parental distress (OR = 1.87, 95%CI = 1.67–2.10), and peer problems (OR = 2.59, 95%CI = 2.23–3.00). Additionally, baseline pain was longitudinally associated with an increased likelihood of suicidality in 2007 (OR = 2.14, 95%CI = 1.77–2.58).

### Class enumeration

**Fig. 3:**
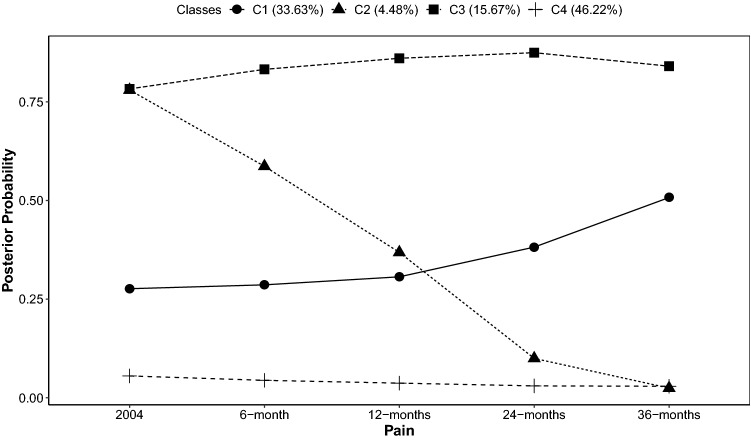
Posterior probability plots for the four-class model, conditioned on age. Classes C1 = increasing; C2 = decreasing; C3 = persistent/recurrent probability of pain and C4: no pain

Model comparisons, based on fit statistics (*n* = 7935; BIC = 28,145; ssBIC = 28,100; LRT = *p* < 0.001), successful convergence (Table S1), class interpretability and size (Fig. 3) identified the four-class conditional model, controlling for age, with the intercept, linear and quadratic slopes as the best fitting model (see Fig. S2): Class one (“Increase”: *n* = 2669; 33.6%) showed an initially low probability of pain, which increased to an approximately equal probability of pain or no pain in 2007. Class two (“Decrease”: *n* = 355; 4.5%) showed an initially high probability of pain, which steeply declined to a low probability of pain in 2007. Class three (“Persistent/Recurrent”: *n* = 1244; 15.7%) showed a persistently high or recurrent probability of pain across time, and class four (“No pain”: *n* = 3667; 46.2%) showed a consistently low probability of pain across time. We identified moderate class separation (entropy = 0.54), driven by the probability that individuals belonging to class two (“Decrease”) may also be classified as belonging to class one (“Increase”; Table S2). Therefore, the decreasing pain trajectory needs to be interpreted with care.

### Predictors of class membership

**Fig. 4:**
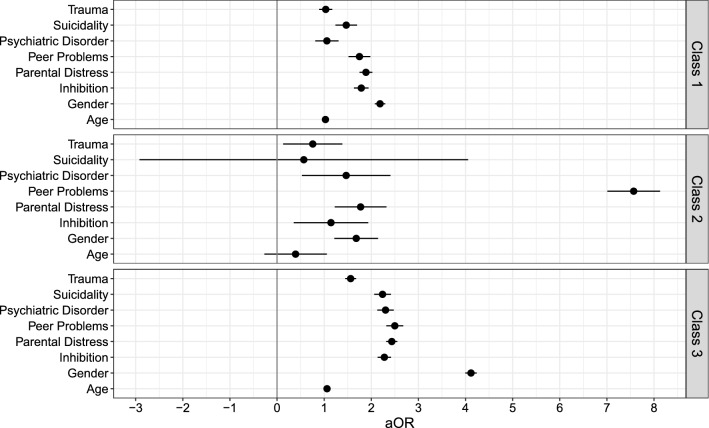
Effects of each predictor on class membership, compared to the ‘no-pain’ trajectory. Class 1 = increasing; Class 2 = decreasing; Class 3 = persistent/recurrent probability of pain. *aOR* adjusted odds ratio

Multinominal, multivariate logistic regression (*n* = 7729; Table S3 and Fig. 4) revealed similar class counts and proportions (Class 1 = 2694 (35%); Class 2 = 334 (4%); Class 3 = 1156 (15%); Class 4 = 3545 (46%)), confirming the stability of the latent class structure. Young people in the increasing pain trajectory (vs. no pain) were twice as likely to be girls (aOR = 2.19; 95%BCI = 1.77–2.76) and to report baseline inhibitory control deficits (aOR = 1.79; 95%BCI = 1.33–2.36), parental distress (aOR = 1.89; 95%BCI = 1.38–2.40) and peer problems (aOR = 1.75; 95%BCI = 1.03–2.67). Young people in the decreasing pain trajectory (vs. no pain) were eight times more likely to report peer problems at baseline (aOR = 7.57; 95%BCI = 1.42–96.90), with no other class differences. Young people with a persistent/recurrent probability of pain (vs. no pain) were four times more likely to be girls (aOR = 4.12; 95%BCI = 3.19–5.14) and they were up to three-times more likely to endorse all clinical correlates at baseline (suicidality (aOR = 2.24; 95%BCI = 1.59–3.26), psychiatric disorder (aOR = 2.30; 95%BCI = 1.65–3.25), inhibitory control deficits (aOR = 2.28; 95%BCI = 1.68–2.94), childhood trauma (aOR = 1.56; 95%BCI = 1.25–2.00), parental distress (aOR = 2.44; 95%BCI = 1.94–2.97) and peer problems (aOR = 2.5; 95%BCI = 1.79–3.64)).

### Distal outcome

Using multinominal logistic regression, we examined the association between class membership and suicidality in 2007, after controlling for baseline suicidality, psychiatric disorder, age and gender (*n* = 7844). Similar class counts and proportions (Class 1 = 2652 (34%); Class 2 = 359 (5%); Class 3 = 1213 (15%); Class 4 = 3620 (46%)) confirmed the latent class structure. Differences in thresholds between the four classes were revealed (*X*^2^(3) = 35.7, *p* < 0.001). The ‘persistent/recurrent’ (vs. no pain) trajectory was associated with an increased risk of suicidality in 2007 (aOR = 1.03, p_(adjusted)_ < 0.001, 95%BCI = 1.01–1.06), after controlling for baseline suicidality, age, gender and psychiatric disorders. Although the ‘increasing’ pain trajectory was associated with a decreased risk (aOR = 0.85, p_(adjusted)_ < 0.001, 95%BCI = 0.54–1.36), the wide confidence intervals include ‘1’, suggesting instability of these results. The ‘decreasing’ (aOR = 0, p_(adjusted)_ = 1.00, 95%BCI = -0.000–0.000) pain trajectory did not differ from the no pain trajectory.

## Discussion

We used longitudinal, population-based data on pain, to establish the number and nature of distinct pain trajectories in 5- to 16-year-olds, and how these trajectories relate to baseline and future suicidality. We identified the following four pain trajectories: an increasing, decreasing, and persistent/recurrent probability of pain, and no pain. These pain trajectories were associated with unique demographic and clinical correlates. However, only the persistent/recurrent (vs. no-pain) trajectory was predicted by baseline suicidality and subsequently predicted future suicidality. These findings provide initial evidence for a potential bidirectional persistent/recurrent pain–suicidality association in young people and emphasise the importance of early prevention and targeted care.

As hypothesised, we found that individual variability in pain can be captured with distinct pain trajectories, as represented by the four-class conditional model, controlling for age (increasing (34%), decreasing (5%), persistent/recurrent pain (16%) and no pain (46%)). The ‘persistent/recurrent’ and ‘no pain’ trajectories align with research on common pain problems throughout adolescence (sporadic = 57%; frequent = 9% and non-to-minimal pain = 34% [18]). Whilst our ‘increasing’ and ‘decreasing’ pain trajectories were previously captured by a single ‘sporadic’ pain trajectory [18], similar trajectory patterns and prevalence rates to our four-class solution have been revealed in a study on lower back pain in adolescence and young adulthood (increase: 22%, decrease: 15%, high: 10% and low: 53% [38]). Thereby, our findings suggest that heterogeneity in pain in UK youth can be captured with four pain trajectories that are largely consistent with the literature.

Crucially, these four pain trajectories were distinctively associated with baseline demographic and clinical correlates, which is consistent with our second hypothesis. Young people in the decreasing pain trajectory (vs. no pain) were almost eight times more likely to report peer problems at baseline with no other differences. As the decreasing pain trajectory was indistinguishable from the ‘persistent/recurrent’ trajectory at baseline, this unexpected finding aligns with research, suggesting peer problems in young people with pain [39]. Yet, this observation needs to be interpreted cautiously given the wide confidence intervals and low entropy.

Girls were twice to four times more likely to be classified into the increasing and persistent/recurrent pain trajectories (vs. no pain), thereby supporting research emphasising the increased risk of pain in girls compared to boys [18, 38]. Both trajectories were characterised by inhibitory control deficits, parental distress, and peer problems, emphasising the importance of these correlates in the development and maintenance of pain. Indeed, theoretical models of chronic pain highlight the importance of inhibitory control in modulating pain, suggesting that young people with early inhibitory control deficits may be unable to divert their attention away from pain, leading to a preoccupation with painful stimuli and an increased risk of recurrent or persistent pain [40, 41]. The present findings also emphasise the need to consider the family environment in the young person’s experience of and response to pain. Parental distress and catastrophising are associated with parental protective behaviours (activity restrictions), which longitudinally, however, may cause more pain and functional disability [42]. Hence, a systemic approach in paediatric pain treatment is crucial.

Baseline childhood trauma and psychiatric disorders were uniquely associated with an increased likelihood of persistent/recurrent compared to no pain. By emphasising childhood trauma and highlighting psychiatric disorders more generally, these findings expand previous research, revealing higher rates of anxiety and depression in young people with more ‘painful’, compared to ‘no-pain’ trajectories [18, 43]. Childhood trauma and psychiatric disorders are often associated with ‘*psychological* pain’ (the persistent, unpleasant and unbearable feeling of perceived self-deficiency or inability [44, 45]). Like physical pain, psychological pain is an independent construct known to be associated with an increased risk of suicidality [45]. Specifically, intense psychological pain, resulting from the perception of thwarted essential needs in the absence of expected change, may be a key risk factor for suicidality [46]. These similarities between physical and psychological pain in terms of future suicidal risk were explained by a shared neurobiology underpinning the experience of both physical and psychological pain [45]. The finding that childhood trauma and psychiatric disorders predicted a persistent/recurrent pain trajectory provides support for the interconnectedness between psychological and physical pain, especially if persistent or recurrent, which underscores the multidimensionality of the pain experience and is consistent with the official definition of pain as both a sensory and an emotional experience [11].

As hypothesised, we found unique relationships between baseline suicidality and an increased risk of future persistent/recurrent pain (vs. no pain), and between persistent/recurrent pain (vs. no pain) and an increased risk of future suicidality, after rigorously controlling for baseline suicidality, age, gender, and psychiatric disorders. Whilst these relationships were non-significant for the decreasing pain trajectory, the increasing pain trajectory was negatively associated with future suicidality—an unstable finding, possibly because the increasing pain trajectory only showed a moderate probability of pain in 2007. Alternatively, young people may be initially more resilient towards the adverse effects of pain, and suicidal risk might increase as pain persists or reoccurs (non-linearity). This finding is notable as little is known about the independent roles of chronic and acute pain, respectively, in the pain-suicidality association [47]. Interestingly, girls were four times more likely to follow the persistent/recurrent pain trajectory. Hence, menstrual pain may be key in the relationship with suicidality, which aligns with previous research [48]. These findings enhance our understanding by providing initial evidence for a bidirectional relationship between suicidality and particularly persistent/recurrent pain, even after controlling for shared correlates. This finding is important as pain and suicidality may reinforce one another, leading to more disability and at its worst mortality, if unrecognised or untreated.

A similar bidirectional relationship has been proposed for chronic pain and depression in young people [19]. As the pain–suicidality association remained significant after controlling for psychiatric disorders, including depression, we propose that the observed bidirectional relationship here may be driven by risk mechanisms, different from those associated with depression, including, e.g., an acquired capacity to enact self-harm through access to lethal means (analgesics [49]).

### Strength and limitations

This study has considerable strengths, including the use of population-based, longitudinal data and validated measures. The statistical rigor (e.g., bootstrapping and control for shared correlates) increases confidence in the findings and their generalisability.

Yet, the following limitations need to be considered: Whilst the item-based assessments allowed us to explore the pain-suicidality association at large-scale, focussing on the most common types of paediatric pain (headaches and abdominal pain [16]), the SDQ [27] item also included ‘sickness’. Yet, agreement between measurement tools (baseline parental reports of stomach/abdominal pains and migraines/severe headaches) suggests convergent validity.

Although this pain item is not well-suited to differentiate between persistent/recurrent or acute pain in the past six months, or characteristics of the pain experience that may drive this association, by using longitudinal data and advanced statistics, we were able to address major limitations of research to date (e.g., lack of control for important correlates [15]) and explore the longitudinal nature of the child’s pain, suggesting that particularly the probability of persistent/recurrent pain is associated with an increased risk of suicidality, which addresses an important gap in the literature [47]. Focussing on a broad age-range allowed us to capture early manifestations of pain and suicidality, and the predictability of future suicidality during a developmental period where these thoughts and behaviours tend to manifest [21]. Yet, by including children, we relied on parental reports. As both pain and suicidality are subjective experiences, actual prevalence rates might be higher due to a lack of parental awareness [26]. Nevertheless, by identifying associations between pain trajectories and suicidality based on these more conservative estimates, we can be confident that the results are likely robust when using child self-report measures.

Attrition between 2004 and 2007 was characterised by higher levels of baseline pain and suicidality. Nonetheless, identified associations based on the healthier, retained sample suggest that findings are likely robust in the full sample.

Moderate class separation suggests that our trajectories may represent an approximation of an underlying nonnormal distribution, or in fact a mixture of true classes and classes representing such nonnormality [50]. However, convergence between our results and previous research [18, 38] increases confidence in our class solution and its replicability.

## Conclusion

This study rigorously and innovatively suggests that distinct pain trajectories longitudinally relate to suicidality and other clinically modifiable correlates in young people aged 5 to 16 years. The use of population-based, longitudinal data, and control for shared correlates of pain and suicidality allowed us to scrutinise this relationship at large-scale over time, as research and clinical knowledge on suicidality in young people with pain is sparse, and the role of acute and chronic pain unclear. Findings revealed unique correlates of pain trajectories; Suicidality appeared as a particular clinical concern in young people with persistent or recurrent pain. If unrecognised or untreated, both conditions may reinforce one another, potentially leading to further disability and at its worst mortality. Future studies should use more comprehensive measures of pain to assess intensity, interference, duration, and multidimensional aspects of the pain experience. Clinically, our findings suggest that regular screening and targeted support of young people with persistent or recurrent pain is crucial, particularly as asking young people about suicidality may reduce future suicidal risk [51].

## Supplementary Information

Below is the link to the electronic supplementary material.

Supplementary file1 (DOCX 103 KB)

## Data Availability

Syntax files are accessible on the Open Science Framework (project name: ‘Pain Trajectories and Suicidality in Young People’). Data are available via the UK Data Archive.
