# Supplementary material for: Pain and suicidality in children and adolescents: a longitudinal population-based study
Source: Eur Child Adolesc Psychiatry. 2022 Mar 2;32(8):1507–17. doi: 10.1007/s00787-022-01963-2 (PMC10326152; doi:10.1007/s00787-022-01963-2)
Supplement: Supplementary file 1 — Supplementary file1 (DOCX 103 KB) [file 787_2022_1963_MOESM1_ESM.docx]

**Supplementary Information**

Pain and Suicidality in Children and Adolescents:

A Longitudinal Population-Based Study

Verena Hinze^1^ (MSc), Anke Karl^2^ (PhD), Tamsin Ford^3^ (PhD), & Bergljot Gjelsvik^1,4^ (PhD)

^1^Department of Psychiatry, University of Oxford, UK

^2^Department of Psychology, University of Exeter, UK

^3^Department of Psychiatry, University of Cambridge, UK

^4^Department of Psychology, University of Oslo, Norway

**Target journal**. European Child & Adolescent Psychiatry

**Correspondence:**

Verena Hinze (ORCID: 0000-0001-7722-2064)

University of Oxford, Department of Psychiatry

Warneford Lane, Oxford, OX3 7JX

Email: verena.hinze@psych.ox.ac.uk

**Table of Content**

|  | **Page** |
| --- | --- |
| **Supplement 1.** Additional Information on Study Design and Measures. | 3-4 |
| **Supplement 2:** Additional Information on the Statistical Analyses. | 5-6 |
| **FigS1**. Prevalence of pain across assessment waves. | 7 |
| **TableS1.** Fit statistics for the class enumeration. | 8 |
| **FigS2.** Comparison of class solutions for the conditional models, with the intercept, linear and quadratic slopes. | 9 |
| **TableS2.** Average latent class probabilities for most likely latent class membership (row) by latent class (column). | 10 |
| **TableS3.** Prediction of class membership (*n*=7729). | 11 |
| **References for the Supplementary Information** | 12 |

**Supplement 1. Additional Information on Study Design and Measures.**

**Study Design**

Secondary data analyses were performed using baseline and follow-up data of the British Child and Adolescent Mental Health Survey [BCAMHS] in 2004 [1] and 2007, with interim assessments at 6-, 12- and 24-months follow-up [2]. In 2004, a representative sample of 5 to 16-year-olds was obtained from the Child Benefit Centre that held centralised records on approximately 90% of young people, living in private households in Great Britain. Exclusion criteria were a) no valid postcode/non-contacts, b) postal sector too small, c) child considered too sensitive for participation, and d) no written informed consent/refusal. In 2004 and 2007, data were collected through standardised face-to-face interviews, which were supplemented with self-report questionnaires for sensitive topics. For the interim assessments brief self-report questionnaires were sent to all parents, except at six-months follow-up, to aid sample retention [1-3]. The 6-month assessment aimed to assess changes in psychopathology over time in children who warranted intervention (*n*=651) compared with their healthier peers (*n*=500) [4].

**Psychiatric Disorder**

The presence or absence of psychiatric disorder was identified using a standardised multi-informant diagnostic interview schedule (Development and Well-Being Assessment [DAWBA] [5]). Structured questions, which directly map onto the diagnostic criteria for the identification of psychiatric disorders in the International Classification of Diseases, 10th Edition (ICD-10 [6]) and the Diagnostic and Statistical Manual of Mental Disorders, Fourth Edition (DSM-IV [7]), were combined with semi-structured probes to assess areas of potential difficulty in detail. These interviews were conducted with parents (N=7977), and adolescents aged 11 years and older (*n*=3344), while a shorter questionnaire was mailed to teachers (*n*=6236). Using a case vignette approach, a team of experienced child mental health specialists combined all available information across informants to assign DSM-IV diagnoses to the child [1], with adequate inter-rater reliability (Cohen’s kappa_(any disorder)_=0.86, SE=0.04 [8]). Furthermore, previous validation studies have revealed excellent discriminant validity in terms of identified psychiatric diagnoses in clinical and community samples, as well as considerable agreement between DAWBA diagnoses and case notes [5]. This integrated information was used to obtain the dichotomised ‘Psychiatric Disorders’ variable, referring to the presence or absence of any psychiatric disorder identified across informants in 2004.

**Childhood Trauma**

The experience of childhood trauma was assessed using the range of potential incidents asked of parents during screening for ‘Post Traumatic Stress Disorder’ with the DAWBA. Parents reported whether their child had experienced severe trauma by selecting all traumatic events that applied from the following list: 1) a bad fire, e.g., being trapped in a burning building; 2) a serious and frightening accident, e.g., being in a bad car or train crash; 3) other disasters, e.g., kidnapping; 4) a severe attack or threat, e.g., by a gang; 5) severe physical abuse that he/she still remembers, 6) sexual abuse, 7) rape, 8) witnessed severe domestic violence, 9) saw family member or friend severely attached or threatened, 10) witnessed a sudden death, suicide, serious accident, heart attack, an overdose, etc. and 11) other severe trauma [1]. Given the severity of these events, we coded this binary ‘Childhood Trauma’ variable as present if at least one event was positively endorsed and as absent if no event was reported in 2004.

**Parental distress**

Parental distress was measured with the ‘General Health Questionnaire’ [GHQ-12] [9]. Previous validation studies have identified high values for sensitivity (76.3%), specificity (83.4%), mean area under the ROC curve (0.88), and Cronbach’s alpha (0.82-0.86), suggesting validity and reliability of the GHQ-12 as a screening instrument of psychological distress over the past two weeks [9]. Parents scored each of the 12 items on a four-point scale, ranging from ‘not at all’ to ‘much more/ less than usual’. Positive items that were answered with ‘less than usual’ and negative items that were answered with ‘much more’ received a score of ‘1’, leading to a total score between 0 (no distress) and 12 (severe distress). Consistent with previous research, a cut-off score of 3 was used to create the dichotomous ‘Parental Distress’ variable, referring to the presence or absence of parental distress in 2004 [1,9].

**Inhibitory Control Deficits & Peer Problems**

The Strengths and Difficulties Questionnaire [SDQ] [10] consists of five subscales with five items each, used to screen for childhood psychopathology in the past six months. The response options include ‘Not true’ (0), ‘Somewhat true’ (1) and ‘Certainly true’ (2) for negative statements or reversed for positive statements. Greater subscale scores indicate larger difficulties on each domain [10]. The SDQ has been proven to be a reliable (Cronbach’s alpha: mean=0.73), between-informant correlation (mean=0.34), test-retest reliability after 4 to 6 months (mean=0.62)) and valid (increased prediction of diagnosed psychiatric disorders (mean odds ratio=15.7 for parental reports) screening tool for childhood psychopathology in the past six months, and subscales have been confirmed with factor analyses [11].

For the assessment of inhibitory control deficits, we used the hyperactivity/ inattention subscale of the SDQ completed by the parents in 2004, with a cut-off score of at least 6 indicating the presence of ‘Inhibitory Control Deficits’ [10,12].

For the assessment of peer problems, we used the peer problem subscale of the SDQ [10] completed by the parents in 2004, with a cut-off score of at least 4 indicating the presence of ‘Peer Problems’ [10,12].

**Supplement 2: Additional Information on the Statistical Analyses.**

**Data exploration**

Descriptive statistics were used to explore participant characteristics (R-packages: *psych* version 2.0.12 [13]; and *Hmisc* version 4.2-2 [14]). Using Fisher’s test, we explored cross-sectional and longitudinal associations between pain and suicidality (R-package: *questionr* version 0.7.4 [15]). We explored differences between young people with and without follow-up data with the Pearson’s Chi-squared test for equal proportions for binary data and the two-sample Wilcoxon test for ordinal data (R-package: *stats*, version 3.6.2 [16]).

**Latent Class Growth Analysis**

Latent Class Growth Analysis [LCGA] is a specific type of finite mixture models, which was used to approximate distinct temporal pain trajectories, and to classify young people into their most likely pain trajectory based on their individual response patterns [17,18]. LCGA leads to homogenous growth trajectories in each class and typically to a larger number of identified classes to fully capture all variation within the data [18], which we considered useful given our binary pain data.

***Class enumeration (step 1 and 2)***

In step 1, Latent Class Growth Models were fitted to the pain class indicators. In step 2, multinominal regression analysis was used to assign each participant fractionally to all classes given their posterior probabilities [18,19]. As we expected that up to five distinct pain trajectories could be recovered with our data (i.e., a) no pain, b) persistent/recurrent pain, c) emerging pain, d) resolving pain and e) inconsistent pain), we fitted models with an increasing number of classes (1-6 classes), comparing the fit between each successive model. By fitting a one-class model, we could test whether a single growth trajectory can approximate our entire study sample or whether at least two distinct latent classes can better reproduce and describe our aggregate data [17]. To estimate which functional form describes the growth trajectories best, we compared models with a) the intercept, b) the intercept & linear slope, and c) the intercept, linear slope & quadratic slope [18]. After identification of the appropriate functional form, we compared the most suitable unconditional model (without correlates) to the respective conditional model (controlling for age at baseline) to account for the potential direct effects of age on the estimated growth factors [18]. All models were estimated, using the robust, full information maximum likelihood [FIML] estimation, which corrects standard errors and chi-square statistics for non-normality, and which we chose given our binary class indicator variables (‘pain’) [20]. This method handles missing data by using all available data to estimate parameters and it computes posterior class probabilities for each participant with available pain data based on the variables that are present [21]. Parameters are estimated through an iterative process, with successful model convergence when the estimated parameters reach the largest likelihood (i.e., a global maximum solution [18]). To avoid inferior local solutions, whereby the model converges on a local maximum or minimum, which may not reflect the truly largest or smallest value under the entire curve, random starting values were used [18]. We chose up to 5000 initial start values to thoroughly explore the likelihood surface, of which the 100 best sets were iterated until convergence (number of initial stage iterations = 300).

Consistent with the literature [22], the optimal model was identified by comparing the following goodness-of-fit criteria: Bayesian Information Criterion [BIC] and the sample-size adjusted Bayesian Information Criterion [ssBIC], with lower values reflecting better model fit. We also evaluated the Lo-Mendell-Rubin Likelihood Ratio Test [LMR test], with a significant test, suggesting better fit of the more complex model compared to the model with one less class [22]. We also considered successful convergence, class interpretability and class size (<1% of the sample, reflecting a potential unstable solution) in the selection of our final model [18]. For the identified class solution, we report the ‘entropy’ – the extent of class separation, with a score near one reflecting good classification [18]. After successful class enumeration the estimated class trajectories were plotted in R, using the packages ‘*MplusAutomation*’ version 0.8 [23] and ‘*ggplot2*’ version 3.3.3 [24].

***Predictors of class membership (step 3a)***

For the investigation of class predictors and distal outcomes, we saved the posterior probabilities and class assignments identified in the previous steps 1 and 2. This unbiased ‘assigned class membership’ variable was then used as a nominal latent class indicator [19]. Using multinominal regression, we estimated whether key demographics (age & gender) and clinical correlates (suicidality, psychiatric disorder, childhood trauma, parental distress inhibitory control deficits and peer problems), all measured in 2004, predicted assigned class membership. Within this model, each predictor is conditioned on the effects of all other predictors, allowing us to establish the unique effects of each predictor. We computed 95% bias-corrected bootstrap confidence intervals to reveal the robustness of the results across 1000 bootstrap samples.

***Distal outcomes of class membership (step 3b)***

Finally, we estimated whether this assigned class predicted our distal outcome suicidality in 2007, after adjustment for baseline suicidality, psychiatric disorder, age, and gender. We again set the parameters of our ‘assigned class’ indicator to reflect the level of inaccuracy associated with the modal class assignment. Furthermore, we computed the Wald-test of parameter constraints to reveal significant differences in the estimated thresholds between the four classes, after controlling for baseline suicidality, psychiatric disorder, age, and gender, which we followed up with pairwise comparisons to establish which classes were significantly different from the ‘no pain’ reference class. To account for three pairwise comparisons, p-values were adjusted using the Bonferroni correction (R-package: *stats*, version 3.6.2 [16]). Furthermore, we computed 95% bias-corrected bootstrap confidence intervals to reveal the robustness of the results across 1000 bootstrap samples.

|  |
| --- |
| Legend: 0=Pain absent 1=Pain present |

**FigS1 Prevalence of pain across assessment waves**

Note. The y-axis is scaled based on the number of families, taking part in the respective assessment wave (2004: *N*=7977; 6-months: *n*=1151; 12-months: *n*=6375; 24-months: *n*=5794; 36-months: *n*=5325). The proportions in the bar-charts reflect the proportion of young people with (1) or without (0) pain of those with available data for that assessment wave.

**TableS1. Fit statistics for the class enumeration.**

| **Unconditional Models** | | | | | | | | |
| --- | --- | --- | --- | --- | --- | --- | --- | --- |
|  |  |  |  |  | **Parsimony**  **Criteria** | | **Clustering Criteria** | **Tests** |
| **Growth trends** | **Classes** | **N** | **Parameter** | **LL** | **BIC** | **ssBIC** | **Entropy** | **LMR** |
| **Intercept only model** | 1 | 7935 | 1 | -15831 | 31671 | 31668 | NA | NA |
|  | 2 | 7935 | 3 | -14155 | 28336 | 28326 | 0.65 | *** |
|  | 3 | 7935 | No convergence | | | | | |
|  | 4 | 7935 | No convergence | | | | | |
|  | 5 | 7935 | No convergence | | | | | |
|  | 6 | 7935 | No convergence | | | | | |
| **Intercept + linear slope** | 1 | 7935 | 2 | -15822 | 31663 | 31656 | NA | NA |
|  | 2 | 7935 | 5 | -14131 | 28307 | 28291 | 0.64 | *** |
|  | 3 | 7935 | 8 | -14067 | 28205 | 28180 | 0.50 | *** |
|  | **4** | **7935** | **9** | **-14047** | **28174** | **28145** | **0.55** | ******* |
|  | 5 | 7935 | No convergence | | | | | |
|  | 6 | 7935 | No convergence | | | | | |
| **Intercept + linear slope + quadratic slope** | 1 | 7935 | 3 | -15817 | 31661 | 31652 | NA | NA |
|  | 2 | 7935 | 7 | -14114 | 28291 | 28268 | 0.64 | *** |
|  | 3 | 7935 | 11 | -14057 | 28212 | 28177 | 0.50 | *** |
|  | 4 | 7935 | 14 | -14032 | 28191 | 28146 | 0.55 | *** |
|  | 5 | 7935 | No convergence | | | | | |
|  | 6 | 7935 | No convergence | | | | | |
| **Conditional Models (controlling for Age)** | | | | | | | | |
|  |  |  |  |  | **Parsimony**  **Criteria** | | **Clustering Criteria** | **Tests** |
| **Growth trends** | **Classes** | **N** | **Parameter** | **LL** | **BIC** | **ssBIC** | **Entropy** | **LMR** |
| **Intercept only model** | 1 | 7935 | 2 | -15806 | 31631 | 31624 | NA | NA |
|  | 2 | 7935 | 4 | -14140 | 28317 | 28304 | 0.64 | *** |
|  | 3 | 7935 | No convergence | | | | | |
|  | 4 | 7935 | No convergence | | | | | |
|  | 5 | 7935 | No convergence | | | | | |
|  | 6 | 7935 | No convergence | | | | | |
| **Intercept + linear slope** | 1 | 7935 | 4 | -15784 | 31603 | 31590 | NA | NA |
|  | 2 | 7935 | 7 | -14097 | 28256 | 28234 | 0.64 | *** |
|  | 3 | 7935 | 10 | -14034 | 28158 | 28127 | 0.50 | *** |
|  | 4 | 7935 | No convergence | | | | | |
|  | 5 | 7935 | No convergence | | | | | |
|  | 6 | 7935 | No convergence | | | | | |
| **Intercept + linear slope + quadratic slope** | 1 | 7935 | 6 | -15777 | 31608 | 31589 | NA | NA |
|  | 2 | 7935 | 10 | -14078 | 28246 | 28214 | 0.64 | *** |
|  | 3 | 7935 | 14 | -14023 | 28171 | 28127 | 0.50 | *** |
|  | **4** | **7935** | **14** | **-14009** | **28145** | **28100** | **0.54** | ******* |
|  | 5 | 7935 | No convergence | | | | | |
|  | 6 | 7935 | No convergence | | | | | |

Note. Legend=Bayesian Information Criterion [BIC]; sample-size adjusted Bayesian Information Criterion [ssBIC]; Lo-Mendell-Rubin Likelihood Ratio Test [LMR test]; *** p<.001.

| **Growth Parameters by Class Solution** |
| --- |
| **** |
| **Mean Growth Trajectories by Class Solution** |
| **** |

**FigS2 Comparison of class solutions for the conditional models, with the intercept, linear and quadratic slopes**

Legend: I=intercept, Q=quadratic slope, S=linear slope. The numbering of the classes is based on the final four-class solution, where the ‘No pain’ trajectory (‘’) was coded as the reference class.

**TableS2. Average latent class probabilities for most likely latent class membership (row) by latent class (column).**

| **Latent Class** | 1. Increase | 2. Decrease | 3. Persistent/  Recurrent | 4. No pain |
| --- | --- | --- | --- | --- |
| 1. | **0.69** | 0.05 | 0.11 | 0.15 |
| 2. | 0.35 | **0.48** | 0.07 | 0.11 |
| 3. | 0.23 | 0.04 | **0.71** | 0.02 |
| 4. | 0.19 | 0.02 | 0.01 | **0.78** |

Legend. Increase=increasing probability to report pain across time, Decrease=decreasing probability to report pain across time, Persistent/Recurrent=persistent/recurrent probability of pain across time, ‘No pain’=low probability to report pain across time.

**TableS3. Prediction of class membership (*n*=7729).**

| **Class 1: “Increase”** | | | | |
| --- | --- | --- | --- | --- |
| **Predictors in 2004** | **aOR** | **SE** | **95% bias-corrected bootstrap confidence interval** | |
|  |  |  | **lower** | **upper** |
| Age | 1.028 | 0.049 | 0.934 | 1.127 |
| Gender (1=boys, 2=girls) | **2.187^***^** | **0.243** | **1.770** | **2.761** |
| Suicidality | 1.468 | 0.336 | 0.948 | 2.253 |
| Psychiatric disorder | 1.058 | 0.262 | 0.606 | 1.691 |
| Inhibitory control deficits | **1.788^***^** | **0.277** | **1.334** | **2.364** |
| Childhood trauma | 1.033 | 0.143 | 0.772 | 1.364 |
| Parental distress | **1.886^***^** | **0.257** | **1.375** | **2.397** |
| Peer problems | **1.748^*^** | **0.406** | **1.029** | **2.666** |
| **Class 2: “Decrease”** | | | | |
| **Predictors in 2004** | **aOR** | **SE** | **95% bias-corrected bootstrap confidence interval** | |
|  |  |  | **lower** | **upper** |
| Age | 0.393 | 0.261 | 0.000 | 0.670 |
| Gender (1=boys, 2=girls) | 1.681 | 0.779 | 0.613 | 7.281 |
| Suicidality | 0.568 | 1.980 | 0.000 | 42.297 |
| Psychiatric disorder | 1.467 | 1.377 | 0.000 | 11.507 |
| Inhibitory control deficits | 1.145 | 0.904 | 0.064 | 4.839 |
| Childhood trauma | 0.758 | 0.477 | 0.000 | 2.623 |
| Parental distress | 1.774 | 0.972 | 0.070 | 5.627 |
| Peer problems | **7.567^***^** | **4.232** | **1.421** | **96.902** |
| **Class 3: “Persistent/Recurrent”** | | | | |
| **Predictors in 2004** | **aOR** | **SE** | **95% bias-corrected bootstrap confidence interval** | |
|  |  |  | **lower** | **upper** |
| Age | 1.060 | 0.049 | 0.960 | 1.165 |
| Gender (1=boys, 2=girls) | **4.118^***^** | **0.495** | **3.188** | **5.140** |
| Suicidality | **2.239^***^** | **0.397** | **1.591** | **3.260** |
| Psychiatric disorder | **2.303^***^** | **0.402** | **1.647** | **3.245** |
| Inhibitory control deficits | **2.277^***^** | **0.324** | **1.677** | **2.936** |
| Childhood trauma | **1.562^***^** | **0.177** | **1.252** | **2.001** |
| Parental distress | **2.435^***^** | **0.284** | **1.937** | **2.973** |
| Peer problems | **2.500^***^** | **0.447** | **1.795** | **3.636** |

Note. Significant adjusted odds ratios [aOR] are highlighted in bold. The no pain trajectory (class 4) was used as the reference class. The 95% bootstrap confidence interval was estimated across 1000 bootstrap samples.

Legend. Increase=increasing probability to report pain across time, Decrease=decreasing probability to report pain across time, Persistent/Recurrent=persistent/recurrent probability of pain across time, ‘No pain’=low probability to report pain across time. ***p<0.001, **p<.01, *p<.05.

**References for the Supplementary Information**

1. Green H, McGinnity A, Meltzer H, Ford T, Goodman R (2005). Mental Health of Children and Young People in Great Britain, 2004. London, UK: The Office for National Statistics.
2. Parry-Langdon N (2008). Three Years on: Survey of the Development and Emotional Well-being of Children and Young People. London, UK: Office for National Statistics.
3. Ford TJ, Vizard T, Sadler K et al (2020). Data Resource Profile: The Mental Health of Children and Young People Surveys (MHCYP). Int J Epidemiol 49:363-364. https://doi.org/10.1093/ije/dyz259.
4. Ford T, Hutchings J, Bywater T, Goodman A, Goodman R (2009). Strengths and Difficulties Questionnaire Added Value Scores: Evaluating effectiveness in child mental health interventions. Br J Psychiatry 194:552-558. https://doi.org/10.1192/bjp.bp.108.052373
5. Goodman R, Ford T, Richards H, Gatward R, Meltzer H (2000). The development and well-being assessment: description and initial validation of an integrated assessment of child and adolescent psychopathology. J Child Psychol Psychiatry41:645-656. https://doi.org/10.1111/j.1469-7610.2000.tb02345.x
6. World Health Organisation (1993). The ICD-10 classification of mental and behavioural disorders: Diagnostic criteria for research. Geneva, Switzerland: World Health Organisation. http://www.who.int/classifications/icd/en/GRNBOOK.pdf
7. American Psychiatric Association (1994). Diagnostic and Statistical Manual of Mental Disorders: DSM-IV (4^th^ ed.). Washington, DC: American Psychiatric Association.
8. Ford T, Goodman R, Meltzer H (2003). The British child and adolescent mental health survey 1999: The prevalence of DSM-IV disorders. J Acad Child Adolesc Psychiatry 42:1203-1211. https://doi.org/10.1097/00004583-200310000-00011.
9. Goldberg DP, Gater R, Sartorius N et al (1997). The validity of two versions of the GHQ in the WHO study of mental illness in general health care. Psychol Med 27:191-197. https://doi.org/10.1017/S0033291796004242.
10. Goodman R (1997). The strengths and difficulties questionnaire: A research note. J Child Psychol Psychiatry 38:581-586. https://doi.org/10.1111/j.1469-7610.1997.tb01545.x.
11. Goodman, R (2001). Psychometric properties of the strengths and difficulties questionnaire. J Acad Child Adolesc Psychiatry 40:1337-1345. https://doi.org/10.1097/00004583-200111000-00015.
12. Youth in Mind (2016). Scoring the SDQ. https://www.sdqinfo.org/py/sdqinfo/c0.py. Accessed 06. April 2020.
13. Revelle W (2020). psych: Procedures for Personality and Psychological Research. R version 2.0.12. Northwestern University, Evanston, Illinois; USA. https://CRAN.R-project.org/package=psych.
14. Harrell FE, Dupont C (2020). Hmisc: Harrell Miscellaneous. R package version 4.2-2.

https://CRAN.R-project.org/package=Hmisc.

1. Barnier J, Briatte F, Larmarange J (2020). Package questionr. R package version 0.7.4. https://juba.github.io/questionr/.
2. R Core Team (2019). R: A language and environment for statistical computing. R version 3.6.2 -- "Dark and Stormy Night". Vienna, Austria: R Foundation for Statistical Computing. https://www.R-project.org/.
3. Bauer DJ. Curran PJ (2003). Distributional assumptions of growth mixture models: Implications for overextraction of latent trajectory classes. Psychol Methods 8:338-363. https://psycnet.apa.org/doi/10.1037/1082-989X.8.3.338.
4. Jung T, Wickrama KAS (2008). An Introduction to Latent Class Growth Analysis and Growth Mixture Modeling. Soc Personal Psychol Compass 2:302-317. https://doi.org/10.1111/j.1751-9004.2007.00054.x.
5. Vermunt JK (2010). Latent Class Modeling with Covariates: Two Improved Three-Step Approaches. Political Anal 18:450-469. https://doi.org/10.1093/pan/mpq025.
6. Muthén LK, Muthén BO (1998-2017). Mplus User’s Guide. Eighth Edition. Los Angeles, USA: Muthén & Muthén.
7. Enders CK, Bandalos DL (2001). The relative performance of full information maximum likelihood estimation for missing data in structural equation models. Struct Equ Model 8:430-457. https://doi.org/10.1207/S15328007SEM0803_5.
8. Nylund KL, Asparoutiov T, Muthen BO (2007). Deciding on the number of classes in latent class analysis and growth mixture modeling: A Monte Carlo simulation study. Struct Equ Model 14:538-569. https://doi.org/10.1080/10705510701575396.
9. Hallquist MN, Wiley JF (2018). MplusAutomation: An R Package for Facilitating Large-Scale Latent Variable Analyses in Mplus. Struct Equ Modeling 25:621-638.

https://doi.org/10.1080/10705511.2017.1402334.

1. Wickham H (2016). ggplot2: Elegant Graphics for Data Analysis. New York, USA: Springer-Verlag.
